# Supplementary material for: Comprehensive Annotation and Functional Exploration of MicroRNAs in Lettuce
Source: Front Plant Sci. 2021 Dec 24;12:781836. doi: 10.3389/fpls.2021.781836 (PMC8739914; doi:10.3389/fpls.2021.781836)
Supplement: Supplementary file 2 [file Data_Sheet_8.ZIP › GSM2230747.plot/Lsa-miR396b_Lsat_1_v5_gn_7_68120.1_3339_TPlot.pdf]

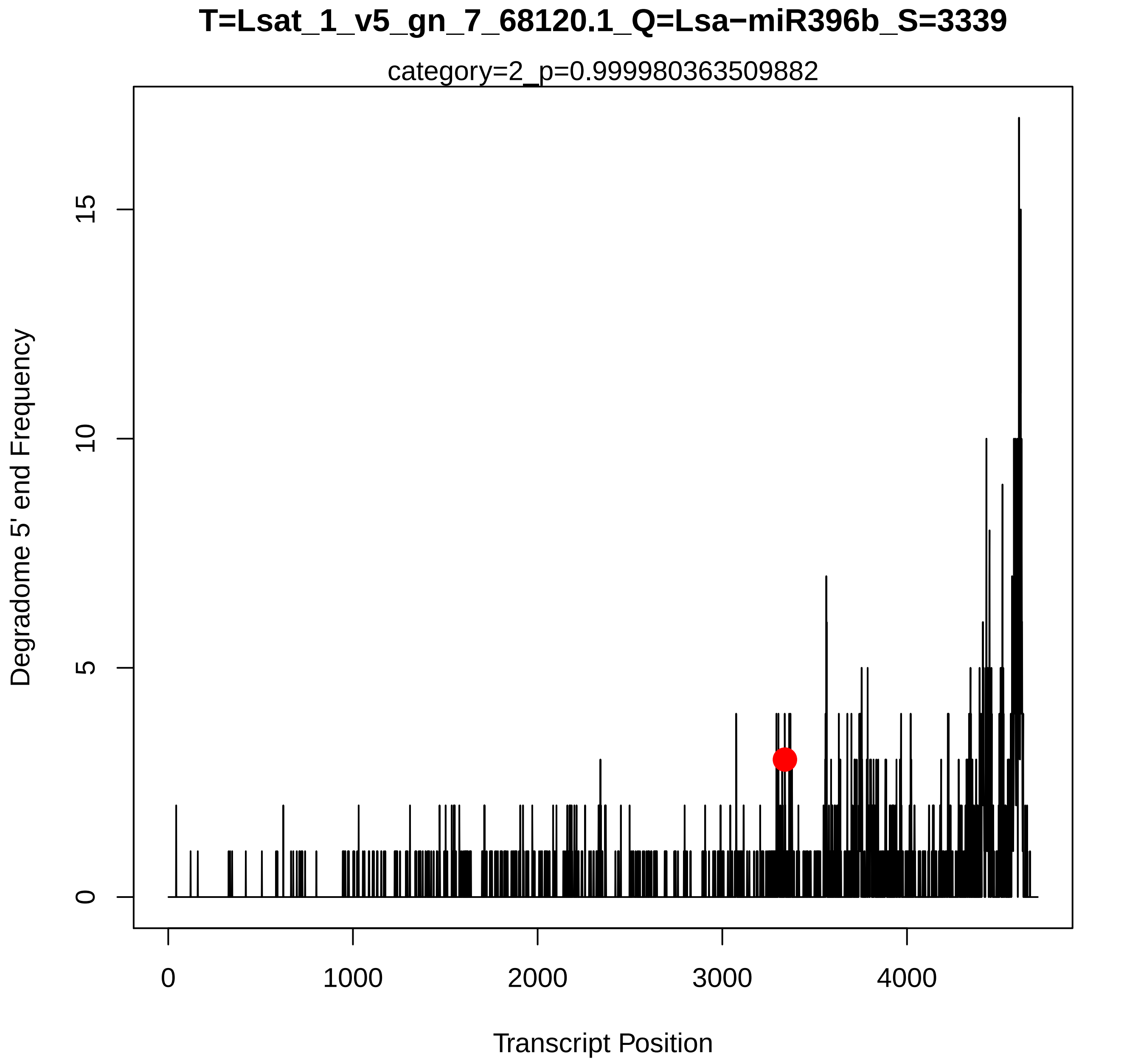

T=Lsat_1_v5_gn_7_68120.1_Q=Lsa−miR396b_S=3339
category=2_p=0.999980363509882
Degradome 5' end Frequency
0
5
10
15
0
1000
2000
3000
4000
Transcript Position
